# Absenteeism and associated labour costs according to depressive symptom severity in the German general population: why preventive strategies matter

**DOI:** 10.1007/s00420-021-01788-7

**Published:** 2021-10-18

**Authors:** Roland Eßl-Maurer, Maria Flamm, Katharina Hösl, Jürgen Osterbrink, Antje van der Zee-Neuen

**Affiliations:** 1 Institute for Nursing Science and Practice, Centre for Public Health and Health Services Research, Paracelsus Medical University, Salzburg, Austria; 2 Institute of General Practice, Family Medicine and Preventive Medicine, Paracelsus Medical University, Salzburg, Austria; 3 Department of Psychiatry and Psychotherapy, Paracelsus Medical University, Nuremberg, Germany

**Keywords:** Absenteeism, Labour costs, Depression, Sick leave, Mental health

## Abstract

**Purpose:**

Depression is a highly prevalent mental health condition with substantial individual, societal and economic consequences. This study focussed on the association of depressive symptom severity with absenteeism duration and employer labour costs.

**Methods:**

Using cross-sectional data from the German Health Update 2014/2015, multivariable zero-inflated Poisson regression (ZIP) models explored the association of depressive symptom severity (8-item depression patient health questionnaire—PHQ-8), with absenteeism weeks during 12 months in men and women working full- or part-time. The predicted sick leave weeks were multiplied by mean average labour costs.

**Results:**

The sample consisted of 12,405 persons with an average sick leave of 1.89 weeks (SD 4.26). Fifty-four % were women and 57% were between 40 and 59 years of age. In men and women, mild, moderate, moderately severe and severe depressive symptoms were associated with a significant factor increase in sick leave weeks compared to persons with no or minimal symptoms. Labour costs increased with increasing symptom severity from € 1468.22 for men with no or minimal depressive symptoms to € 7190.25 for men with severe depressive symptoms and from € 1045.82 to € 4306.30 in women, respectively.

**Conclusion:**

The present results indicate that increasing depressive symptom severity is associated with increasing absenteeism and employer costs. They emphasize the need for implementation, realignment or extension of professional work-site health promotion programmes aiming at the improvement and maintenance of employee health and the reduction of labour costs associated with depression-related sick leave.

**Supplementary Information:**

The online version contains supplementary material available at 10.1007/s00420-021-01788-7.

## Introduction

Depression is one of the most common and consequential mental illnesses (Murray et al. 2013) and is estimated to affect 350 million people worldwide (Marcus et al. 2012). In addition to the large prevalence of depression, it is responsible for the largest global disease burden, accounting for 7% of disability adjusted life years (DALYs, i.e. years of life lost through premature mortality and years lost due to disability for persons suffering from (the consequences of) a health condition) (Ferrari et al. 2013). It is predicted, that by the year 2030, depression will be one of the three leading causes of the global disease burden (Mathers and Loncar 2006). Depression is diagnosed according to the criteria of the ICD-10 (International Statistical Classification of Diseases and Related Health Problems) classification system. The main symptoms of depression include depressed mood, loss of interest or pleasure, and decreased drive and increased fatigability. Additional symptoms of depression include concentration problems, decreased self-esteem, feelings of guilt or worthlessness, negative outlook on the future, suicidal thoughts or actions, sleep disturbances, and loss of appetite. According to the number, type and severity of symptoms and the degree of impairment in social and occupational functioning, the ICD-10 distinguishes between mild, moderate and severe episodes. For a diagnosis of depression, symptoms must have lasted for at least 2 weeks (DIMDI 2019). While the individual impact of depression is tremendous, the substantial economic consequences must be considered as well.

Depression and anxiety disorders cost the global economy an estimated US$1 trillion per year (WHO 2019). Costs of depression include direct costs through healthcare utilization and indirect costs attributable to decreased worker productivity. The prevalence of depression in persons contributing to the workforce is considerable and is associated with decreased work productivity including absenteeism (Henderson and Madan 2014; Musarezaie et al. 2014). In Germany, employers are legally obliged to continuous payment of salary for a maximum period of six consecutive weeks in case of sickness-related absenteeism resulting in excess labour costs without employee output during episodes of sick leave (Federal Government Commissioner for Migration, Refugees and Integration 2020).

However, little is known about the association of depressive symptom severity and duration of absenteeism. Chow et al. (2019) suggested that increasing symptom severity is adversely associated with work productivity. Yet, these findings were based on data originating from the United States of America (USA). To our knowledge, no comparable studies exist for any of the European countries.

While it seems plausible to assume that results will be comparable between the USA and European countries, it must be considered that country-specific features such as economic wealth, social security, human development or geographical region have a relevant influence on work outcomes (van der Zee-Neuen et al. 2017; Chow et al. 2019). Therefore, results should be validated on a national level. The current study aims to shed light on the association of depressive symptom severity and absenteeism as well as on labour costs associated with the duration of sick leave in a representative sample of the general German population. It is hypothesized that (1) absenteeism is increasing with increasing depressive symptom severity and (2) employer labour costs for employees on sick leave raise substantially with increasing symptom severity.

## Methods

Secondary data analyses were carried out using data from the German Health Update (GEDA 14/15) (Robert Koch Institute 2015). The GEDA is a cross-sectional survey among the German-speaking adult population aged 18 and older in Germany that is conducted regularly by the Robert Koch Institute as part of the nationwide health monitoring. The GEDA 14/15 was the fourth wave of the GEDA study and was conducted from November 2014 to July 2015 in a sequential mixed-mode design. To represent the target population, a two-stage stratified random sample was drawn. Study participants could take part in the survey via standardized online questionnaire or via standardized written-postal questionnaire. The exact study design and sampling method is described elsewhere (Lange et al. 2017).

All analyses for the current study were performed using STATA 12.0.

### Outcome variable

Absenteeism/sick leave was included as outcome variable containing information on the number of weeks absent from work due to sickness during 12 months prior to undertaking the GEDA survey. The variable was calculated based on absenteeism days reported in response to the question “During the past 12 months, how many days in total have you been absent from work due to illness?” Measuring absenteeism through self-report with a 12-month recall period can be considered a valid method. In accordance with previous studies, Ferrie et al. (2005) found that the agreement between self-reported sickness absence with absences recorded in employer’s registers was acceptable and that the association between annual self-reported sickness absence days and self-rated health was comparable to those for recorded absence days (Rees and Cooper 1993; Burdorf et al. 1996; Ferrie et al. 2005).

### Covariates and potential confounders

#### Main independent variable

The eight-item depression patient health questionnaire (PHQ-8) is a self-administered screening tool for the detection of depressive symptoms. It is based on eight of nine criteria of the Diagnostic and Statistical Manual of Mental Disorders—Fourth Edition (DSM‐IV) (Kroenke and Spitzer 2002). The ninth criterion was omitted in the data collection due to its sensitive nature (i.e. the item asks about suicidal thoughts). Omission of the ninth criterion has no impact on the validity of the questionnaire (Kroenke et al. 2009; Shin et al. 2019).

The eight items of the PHQ-8 are scored on a four-point scale, indicating how often one has been bothered by problems during a 2-week period prior to undertaking the survey (0 = not at all, 1 = several days, 2 = half the days, 4 = nearly every day). Items focus on (1) interest and pleasure in doing things, (2) feeling down depressed or hopeless, (3) trouble falling or staying asleep or sleeping too much, (4) feeling tired or having little energy, (5) poor appetite or overeating, (6) feeling bad about oneself, (7) trouble concentrating and (8) restlessness or unusual tranquillity. The PHQ-8 was used as the main independent variable (categorical: five categories, 1 = no/minimal depressive symptoms, sum-score PHQ-8: 0–4, 2 = mild depressive symptoms, sum-score PHQ8: 5–9, 3 = moderate depressive symptoms, sum-score PHQ-8: 10–14, 4 = moderately severe depressive symptoms, sum-score PHQ-8: 15–19, 5 = severe depressive symptoms, sum-score PHQ-8: 20–24).

Of the characteristics included in the GEDA dataset, age, sex, body mass index, smoking status, marital status, socioeconomic status, number of comorbidities, social support, physical activity, alcohol consumption and support/care of others are considered relevant factors for the development or worsening of depression throughout the literature and were therefore included in the analyses as potential confounders or covariates (Blazer 2003; Jacobi et al. 2004; Sareen et al. 2006; Marengoni et al. 2011; Kleppang et al. 2018). Below, an overview of these variables and their metric properties is provided.

Demographics

- Age (categorical: 1 = 18–39 years, 2 = 40–59 years, 3 ≥ 60 years).
- Sex (categorical: 1 = male/2 = female).
- Marital status (categorical: 1 = single, 2 = married/living with partner, 3 = widowed, 4 = divorced/separated).
- Socioeconomic status (SES, categorical: 1 = low, 2 = average, 3 = high). The SES-Index was part of the data provided to the authors for further analyses. It reflects a multidimensional sum score based on information on education, profession and income. For the calculation of the variable that was provided to and used by the authors, the three dimensions were first converted to metric scales. Next, they were entered in the SES-Index using equal weights (Lampert et al. 2013).

Health behaviour

- Smoking status (categorical: 1 = daily, 2 = sometimes; 3 = no, cessation, 4 = never smoked).
- Alcohol consumption (categorical: 1 = daily, 2 = weekly, 3 = monthly, 4 = less than monthly, 5 = quit consumption, 6 = never).
- Physical activity (categorical: 1 = inactive, 2 = active during leisure time, 3 = active during work, 4 = active during work and leisure time).

Health status

- Body mass index (BMI, categorical: underweight BMI < 18.5, normal BMI ≥ 18.5 and  ≤ 24.99, overweight ≥ 25 and  < 30, obese ≥ 30).
- Number of comorbidities (continuous: disease count in the past 12 months prior to undertaking the GEDA-survey: excluding depression, including: asthma, chronic bronchitis, myocardial infarction, coronary heart disease, heart insufficiency, osteoporosis, rheumatoid arthritis, osteoarthritis, stroke, diabetes, chronic kidney disease, cirrhosis of the liver, other liver disease, ulcerative colitis, stomach ulcer).

Social/relational

- Support of/care for other persons (categorical: 1 = yes, 2 = no).
- Social support (Oslo-3 Social Support Scale sum-score, 1 = little support, 2 = moderate support, 3 = strong support). The Oslo-3 Scale generates information on (a) the number of persons the respondent can rely on in case of severe, personal problems, (b) the amount of interest and sympathy others have for the respondent and (c) how easy it is to receive practical support from a neighbour. The indicator used in the current study is based on the calculation of three categories reflecting the value ranges of the total point value of the three items asked (Meltzer 2003; Kilpeläinen et al. 2008).

### Statistical analyses

For the purpose of the current cross-sectional analyses. only subjects who indicated to work part- or full-time and without missing values in any of the considered variables were included. First, descriptive characteristics were used to characterize the sample as a total (i.e. including all subjects with part- or full-time work) and by depressive symptom severity. Next, due to overdispersion of zeros in the outcome variable (i.e. many persons did report zero sick leave), zero-Inflated Poisson regression analyses were performed.

Zero-inflated Poisson regression (ZIP) is used for the handling of count data (e.g. weeks of absenteeism) and is particularly suitable for situations in which the number of zero counts is high (e.g. many subjects with no absenteeism). ZIP models assume that excess zeros occur due to a separate process than the actual counts and should be modelled independently.

A ZIP model therefore consists of two parts, a Poisson count part and a logit part. The first part predicts the factor increase in the outcome variable (i.e. sick leave duration in terms of weeks), and the latter part predicts the log odds of excess zeros (Lambert 1992; Cameron and Trivedi 2013; Date 2020). To verify that the response variable was captured better by ZIP than by a regular Poisson regression, Vuong’s test was used.

To determine the final model, manual forward selection was employed for the count part of the model after univariable analyses. Variables which were found to have a significant association with the outcome in univariable analyses remained in the model if they confounded the association of the independent variable of interest with the outcome (i.e. ≥ 10% change in coefficient for PHQ-8) or contributed to the overall model fit estimated by log-likelihood ratio tests. For the latter, significance was assumed at *p* ≤ 0.05 and Chi^2^ changes ≥ 20 were considered relevant. A variance inflation factor analysis was performed on a simplified model (i.e. a linear regression model including the variable of the ZIP count part) to rule out multicollinearity.

Variables in the logit (i.e. inflate) part of the model were selected based on their potential role in increasing the probability of zero sick leave and were retained in the model if they contributed significantly to this probability. Considered variables included:

- Depressive symptom severity, assuming that the absence of depressive symptoms is likely to result in zero sick leave and that higher symptom severity results in smaller log odds of having no sick leave.

- Age, as younger individuals have lower risk of sick leave.

- SES since lower SES is associated with lower risk of sick leave.

- Number of comorbidities since the absence of any (co-)morbidity is likely to result in a higher probability of zero sick leave.

A two-way interaction term including sex and depressive symptom severity was computed and added to the final multivariable ZIP model to explore potential effect modification through sex. Finally, sick leave weeks for each symptom severity stage and sex were predicted.

Based on the final, fully adjusted models, weeks of sick leave for each symptom severity stage were predicted using the Stata postestimation command “margins”.

### Calculation of labour costs

Average weekly labour costs for men and women in full- or part-time employment were retrieved from the statistical yearbook of the Federal German Statistical Office (Statistisches Bundesamt DESTATIS 2019).

Appropriate values (i.e. € 853.47 for women working full-time and € 491.83 for women working part-time/€ 1041.84 for men working full-time and € 539.71 for men working part-time) were assigned to the study subjects. Sick leave weeks predicted by the ZIP model were then multiplied with the average labour costs for men and women, respectively.

## Results

The full sample including persons with part- or full-time work consisted of 12,405 subjects of whom 8910 (71.83%) worked full-time, 6605 (53.42%) were women, 7070 (56.99%) were between 40 and 59 years of age, 6081 (49.02%) had a normal BMI and 7119 (57.39%) were married or in a relationship and living together. The average sum of sick leave in 1 year was 1.89 (SD 4.26) weeks. A more extensive description of the characteristics of the sample as a total and by depressive symptom severity is provided in Table 1.

**Table 1:** Characteristics of the study population as a total and by depressive symptom severity

	Total (*N* = 12,405)	No/minimal depressive symptoms^a^ (*n* = 8573)	Mild depressive symptom^b^ (*n* = 2864)	Moderate depressive symptoms^c^ (*n* = 704)	Moderately severe depressive symptoms^d^ (*n* = 214)	Severe depressive symptoms^e^ (*n* = 50)
Full-time employment, *n* (%)	8910 (71.83)	6313 (73.64)	1955 (68.26)	470 (66.76)	142 (66.36)	30 (60.00)
Weeks sick leave past 12 months, mean (SD)	1.89 (4.26)	1.41 (3.15)	2.40 (4.83)	4.06 (7.63)	5.25 (8.25)	9.08 (11.55)
Age, *n* (%)						
18–39 years	4477 (36.09)	2990 (34.88)	1117 (39.00)	265 (37.64)	90 (42.06)	15 (30.00)
40–59 years	7070 (56.99)	4933 (57.54)	1593 (55.62)	400 (56.82)	113 (52.80)	31 (62.00)
≥ 60 years	858 (6.92)	650 (7.58)	154 (5.38)	39 (5.54)	11 (5.14)	4 (8.00)
Sex, female	6605 (53.42)	4285 (49.98)	1699 (59.32)	455 (64.63)	131 (61.21)	35 (70.00)
Body mass index						
Underweight, < 18.5	204 (1.64)	122 (1.42)	56 (1.96)	20 (2.84)	4 (1.87)	2 (4.00)
Normal weight, 18.5–24.9	6081 (49.02)	4276 (49.88)	1396 (48.74)	304 (43.18)	85 (39.72)	20 (40.00)
Overweight, 25–29.9	4148 (33.44)	2959 (34.52)	893 (31.18)	219 (31.11)	58 (27.10)	19 (38.00)
Obesity, ≥ 30	1972 (15.90)	1216 (14.18)	519 (18.12)	161 (22.87)	67 (31.31)	9 (18.00)
Smoking status, *n* (%)						
Daily	2445 (19.71)	1504 (17.54)	653 (22.80)	206 (29.26)	63 (29.44)	19 (38.00)
Occasionally	842 (6.79)	554 (6.46)	224 (7.82)	47 (6.68)	15 (7.01)	2 (4.00)
Quit smoking	3627 (29.24)	2512 (29.30)	830 (28.98)	203 (28.84)	67 (31.31)	15 (30.00)
Never smoked	5491 (44.26)	4003 (46.69)	1157 (40.40)	248 (35.23)	69 (32.24)	14 (28.00)
Marital status, *n* (%)						
Single	4020 (32.41)	2644 (30.84)	1020 (35.61)	261 (37.07)	79 (36.92)	16 (32.00)
Married/living with partner	7119 (57.39)	5139 (59.94)	1519 (53.04)	335 (47.59)	102 (47.66)	24 (48.00)
Widowed	150 (1.21)	105 (1.22)	31 (1.08)	11 (1.56)	3 (1.40)	0 (0.00)
Divorced/separated	1116 (9.00)	685 (7.99)	294 (10.27)	97 (13.78)	30 (14.02)	10 (20.00)
Socioeconomic-status, *n* (%)						
Low	1325 (10.68)	828 (9.66)	322 (11.24)	121 (17.19)	47 (21.96)	7 (14.00)
Average	6741 (54.34)	4511 (52.62)	1652 (57.68)	428 (60.80)	118 (55.14)	32 (64.00)
High	4339 (34.98)	3234 (37.72)	890 (31.08)	155 (22.02)	49 (22.90)	11 (22.00)
Number of comorbidities, mean (SD)	0.27 (0.63)	0.21 (0.52)	0.36 (0.72)	0.52 (0.90)	0.61 (0.96)	0.86 (1.50)
Oslo-3 social support scale, *n* (%)						
Little support	1823 (14.70)	886 (10.33)	595 (20.78)	222 (31.53)	92 (42.99)	28 (56.00)
Moderate support	6886 (55.51)	4730 (55.17)	1670 (58.31)	369 (52.41)	101 (47.20)	16 (32.00)
Strong support	3696 (29.79)	2957 (34.49)	599 (20.91)	113 (16.05)	21 (9.81)	6 (12.00)
Physical activity, *n* (%)						
Inactive	3588 (28.92)	2389 (27.87)	872 (30.45)	242 (34.38)	70 (32.71)	15 (30.00)
Active during leisure time	3592 (28.96)	2678 (31.24)	721 (25.17)	138 (19.60)	43 (20.09)	12 (24.00)
Active during work	2879 (23.21)	1839 (21.45)	732 (25.56)	231 (32.81)	64 (29.91)	13 (26.00)
Active during work and leisure time	2346 (18.91)	1667 (19.44)	539 (18.82)	93 (13.21)	37 (17.29)	10 (20.00)
Alcohol consumption, *n* (%)						
Daily	957 (7.71)	639 (7.45)	234 (8.17)	53 (7.53)	26 (12.15)	5 (10.00)
Weekly	5519 (44.49)	3983 (46.46)	1190 (41.55)	251 (35.65)	73 (34.11)	22 (44.00)
Monthly	3303 (26.63)	2262 (26.39)	793 (27.69)	187 (26.56)	53 (24.77)	8 (16.00)
Less than monthly	1628 (13.12)	1078 (12.57)	384 (13.41)	129 (18.32)	33 (15.42)	4 (8.00)
Quit consumption	333 (2.68)	189 (2.20)	97 (3.39)	30 (4.26)	11 (5.14)	6 (12.00)
Never	665 (5.36)	422 (4.92)	166 (5.80)	54 (7.67)	18 (8.41)	5 (10.00)
Support of/care for other persons (yes), *n* (%)	1968 (15.86)	1290 (15.05)	470 (16.41)	150 (21.31)	46 (21.50)	12 (24.00)

^a^Sum-score PHQ-8: 0–4

^b^Sum-score PHQ-8: 5–9

^c^Sum-score PHQ-8: 10–14

^d^Sum-score PHQ-8: 15–19

^e^Sum-score PHQ-8: 20–24

### Association of depressive symptom severity with sick leave

Vuong’s test confirmed that the ZIP model was superior to a regular Poisson regression model. Results of the log-likelihood ratio tests during the model building process and estimated association of symptom severity with sick leave for each step in the model building process are presented in Supplementary Table S1.

The final model included the following covariates in the count part of the model: age, sex, BMI, smoking status, marital status, SES and number of comorbidities. The logit part of the model entailed depressive symptom severity, age, SES and number of comorbidities. Alcohol consumption was removed from the model due to multicollinearity.

The interaction term of sex with depressive symptom severity was significantly associated with absenteeism. Therefore, stratified analyses based on sex were performed.

Compared to men and women with no or minimal depressive symptoms, men and women with mild, moderate, moderately severe and severe depressive symptoms had a significantly higher number of sick leave weeks. In men, the number of sick leave weeks increased by a factor of 1.33 [1.27;1.40] for mild depressive symptoms, 1.61 [1.50;1.73] for moderate depressive symptoms, 1.91 [1.71;2.13] for moderately severe depressive symptoms and 3.84 [3.29;4.50] for severe depressive symptoms.

In women, the number of sick leave weeks increased by factor 1.37 [1.31;1.43] for mild, 2.04 [1.93;2.15] for moderate, 2.54 [2.35;2.75] for moderately severe and 2.96 [2.63;3.33] for severe depressive symptoms. Table 2 shows the univariable and multivariable results of the ZIP analyses.

**Table 2:** Adjusted association of depressive symptom severity with weeks of sick leave during 12 months in men and women with full- and part-time employment (*n* = 12,405)

Count part of zero-inflated Poisson model				
	Exp (*B*) [95%CI]			
	Men		Women	
	Univariable	Multivariable	Univariable	Multivariable
PHQ-8 (depressive symptoms, reference = no/minimal)				
Mild	1.51 [1.44;1.59]	1.33 [1.27;1.40]	1.42 [1.37;1.49]	1.37 [1.31;1.43]
Moderate	2.11 [1.96;2.26]	1.61 [1.50;1.73]^a^	2.37 [2.25;2.50]	2.04 [1.93;2.15]
Moderately severe	2.43 [2.19;2.71]	1.91 [1.71;2.13]	3.03 [2.81;3.27]	2.54 [2.35;2.75]
Severe	5.34 [4.58;6.23]	3.84 [3.29;4.50]	4.02 [3.58;4.51]	2.96 [2.63;3.33]
Age group (reference = 18–39 years)				
40–59 years	1.65 [1.57;1.74]	1.35 [1.28;1.43]	1.61 [1.55;1.68]	1.41 [1.34;1.47]
≥ 60 years	3.21 [2.99;3.44]	2.40 [2.22;2.59]	2.04 [1.90;2.19]	1.61 [1.49;1.75]
BMI (reference = normal weight)				
Underweight	0.48 [0.30;0.77]	0.56 [0.35;0.90]	0.83 [0.72;0.94]	0.82 [0.72;0.93]
Overweight	1.29 [1.23;1.35]	1.14 [1.08;1.19]	1.45 [1.39;1.51]	1.26 [1.21;1.31]
Obesity	1.72 [1.63;1.82]	1.24 [1.17;1.31]	1.63 [1.55;1.70]	1.26 [1.20;1.32]
Smoking status (reference = never smoked)				
Daily	1.78 [1.68;1.88]	1.43 [1.35;1.51]	1.33 [1.27;1.39]	1.08 [1.03;1.13]
Occasionally	0.97 [0.89;1.07]	0.99 [0.91;1.09]	0.78 [0.72;0.85]	0.80 [0.73;0.87]
Quit smoking	1.66 [1.58;1.75]	1.30 [1.23;1.37]	1.15 [1.10;1.20]	1.01 [0.96;1.05]
Marital status (reference = single)				
Married/living with partner	1.34 [1.28;1.41]	1.07 [1.01;1.13]	1.21 [1.16;1.26]	0.98 [0.93;1.02]
Widowed	2.15 [1.73;2.68]	1.36 [1.09;1.70]	2.21 [2.00;2.44]	1.37 [1.23;1.52]
Divorced/separated from partner	1.92 [1.77;2.07]	1.38 [1.27;1.49]	1.53 [1.45;1.62]	1.08 [1.02;1.15]
Socioeconomic-status (reference = high)				
Low	2.38 [2.22;2.56]	1.78 [1.66;1.91]	1.76 [1.65;1.87]	1.28 [1.20;1.37]
Medium	2.08 [1.97,2.21]	1.83 [1.73;1.94]	1.39 [1.33;1.45]	1.22 [1.16;1.27]
Number of comorbidities	1.46 [1.43;1.49]	1.24 [1.21;1.26]	1.38 [1.36;1.41]	1.18 [1.15;1.20]

Logit part of zero-inflated multivariable Poisson model		
	B [95%CI]	
	Men	Women
PHQ-8 (depressive symptoms, reference = no/minimal)*		
Mild	−0.49 [−0.67;−0.31]	−0.41 [−0.56;−0.26]
Moderate	−0.94 [−1.31;−0.57]^b^	−0.74 [−1.01;−0.47]
Moderately severe	−0.75 [−1.36;−0.14]	−0.82 [−1.29;−0.35]
Severe	−0.91 [−2.25;0.43]	−1.82 [−3.03;−0.61]
Age group (reference = 18–39 years)		
40–59 years	0.77 [0.59;0.96]	0.99 [0.81;1.16]
≥ 60 years	1.91 [1.65;2.17]	1.60 [1.34;1.86]
Socioeconomic-status (reference = high)		
Low	0.69 [0.46;0.93]	0.79 [0.58;1.01]
Medium	0.45 [0.28;0.63]	0.28 [0.13;0.43]
Number of comorbidities	−0.31 [−0.42;−0.20]	−0.30 [−0.40;−0.19]

^*^More severe depressive symptoms result in smaller log odds of certainly having zero sick leave weeks

^a^Interpretation example: Compared to men with no or minimal depressive symptoms, men with moderate depressive symptoms have a 1.61 times higher number of sick leave weeks

^b^Interpretation example: Compared to men with no or minimal depressive symptoms, the log odds of certainly having zero sick leave weeks are −0.94 in men with moderate depressive symptoms

Based on the fully adjusted ZIP models, the predicted weeks of sick leave were 1.46 [95% CI 1.41;1.51] in men with no/minimal depressive symptoms, 2.25 [95% CI 2.13;2.37] in men with mild depressive symptoms, 3.02 [95% CI 2.74;3.29] in men with moderate depressive symptoms, 3.43 [95% CI 2.87;3.99] in men with moderately severe depressive symptoms and 7.15 [95% CI 5.00;9.30] in men with severe depressive symptoms.

**Fig. 1:**
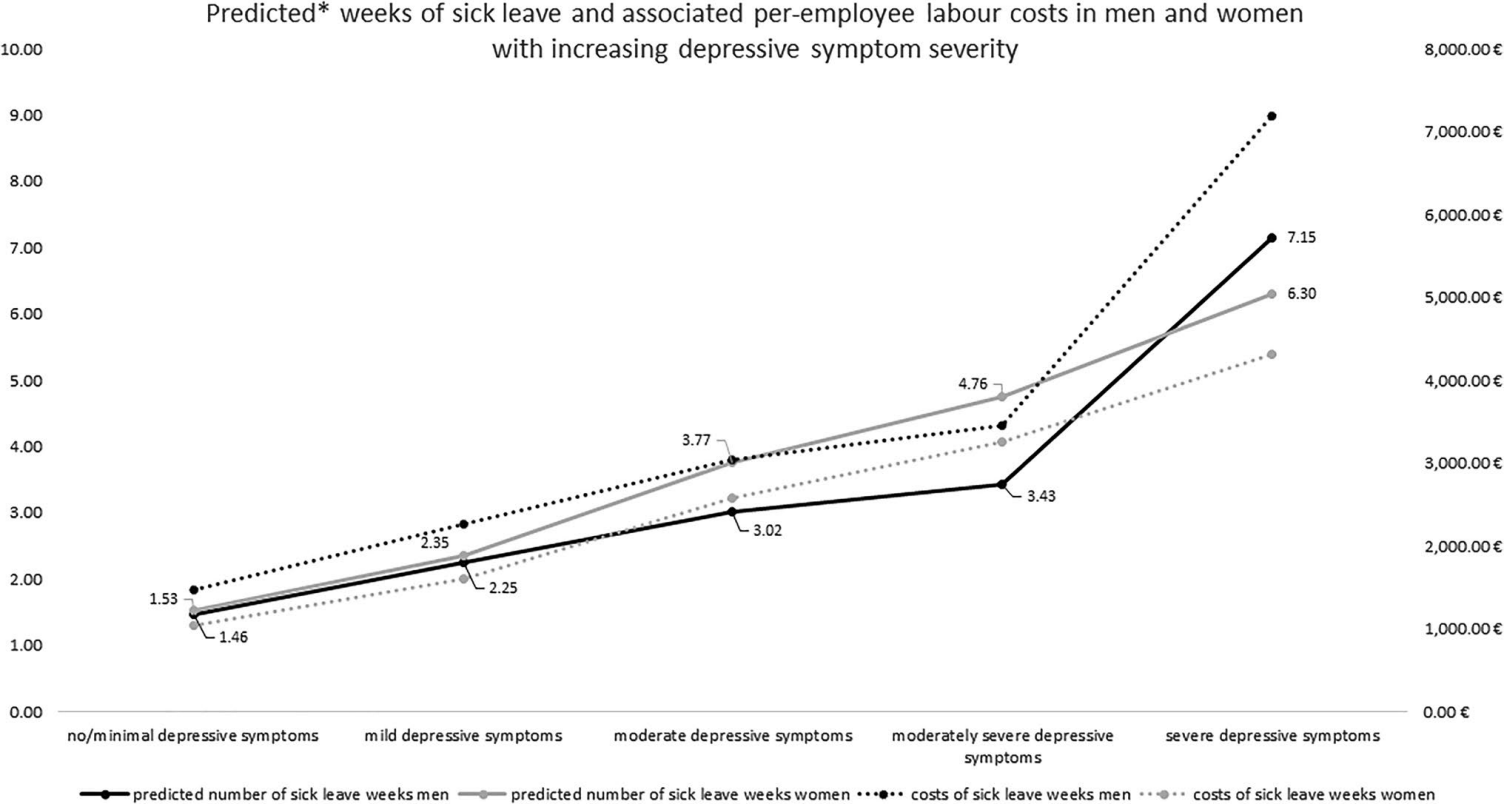
Predicted weeks of sick leave and associated per employee labour costs in men and women with increasing depressive symptom severity

In women with no/minimal, mild, moderate, moderately severe and severe depressive symptoms, the predicted weeks of sick leave were 1.53 [95% CI 1.48;1.58], 2.35 [95% CI 2.25;2.50], 3.77 [95% CI 3.53;4.01], 4.76 [95% CI 4.23;5.29] and 6.30 [95% CI 5.35;7.26], respectively (Fig. 1).

### Labour costs

The average weekly labour costs in the study sample were €1764.94 (SD 1277.62) for men and €1,370.99 (SD 914.54) for women. Costs of sick leave according to depressive symptom severity in men were €1486.22 for no/minimal symptoms, €2262.67 for mild symptoms, €3037.00 for moderate symptoms. €3449.31 for moderately severe symptoms and €7190.25 for severe symptoms. In women, these costs were €1045.82; €1606.32; €2576.95; €3253.65 and €4306.30, respectively (Fig. 1).

## Discussion

The present study showed that sick leave and costs for employers of persons with depressive symptoms rise substantially with increasing, self-reported symptom severity. To our knowledge, this is the first study focussing on the association of depressive symptom severity with absenteeism and related costs in the European region. However, the present findings are in line with other international studies confirming that absenteeism in employees with depressive symptoms is larger than in those without and that the severity of depression adversely affects their workability (Lerner et al. 2010; Beck et al. 2011; Johnston et al. 2019).

In agreement with the current study findings, Mcternan et al. (2013) reported that sick leave costs in employees with mild depressive symptoms are notably higher than in employees with no or minimal depressive symptoms. Moreover, we found that an increasing severity of symptoms was associated with an increasing risk of sick leave duration.

These results apply to both men and women.

Interestingly, the rather continuous increase in predicted sick leave duration and associated costs in men was substantially steeper when comparing the moderately severe symptom category to the severe symptom category.

A potential explanation might be that men are more likely to continue work until their health fully prevents work continuation. On the other hand, considering the cross-sectional nature of our analyses, the phenomenon might indicate that absence from work is related to an increased symptom severity. An underlying mechanism could be a decreased sense of self-esteem related to labour market inactivity, which is related to more severe symptoms of depression in men but not in women (Alvaro et al. 2019).

The large study sample, the lengthy assessment period of work absenteeism, comprehensiveness and quality of available data as well as their analyses pronounce the strength of the present study. However, it must also be considered that the cross-sectional data collection resulted in the recording of all variables at the same time with the same methodology. Therefore, there is a risk for the occurrence of same-source bias (i.e. people with pronounced depressive symptoms might have assessed other variables, like absenteeism more negatively). This might have resulted in an overestimation of the adverse association of depressive symptom severity and absenteeism.

As in all cross-sectional studies, no conclusions on the causality of observed associations can be drawn. While it seems plausible to assume that higher levels of depressive symptoms lead to an increased degree of absenteeism, being absent from work could also lead to social isolation and, in extension, to higher levels of depression. However, both directions would indicate a need for measures aiming at the prevention of depression at or initiated by the work place. Here, the subjectively perceived health risk through work, that appeared to be most pronounced in individuals with severe depression symptoms in our study, should be taken into account. Since individuals’ health and work beliefs are known to influence individual sickness absence decisions Sallis and Birkin 2014), there is a need for specific rehabilitation strategies with person-centred approach (UWV 2020).

The external validity of the current study results is strong for the German context, which is mainly attributable to the adequate sampling strategy used for the collection of data. In Germany, salary payment in case of sickness absence continues for a period of up to six subsequent weeks for each cause of sickness. Additional payment continuation for the same cause of sick leave may be required if a period of ≥ 6 months lies between the two periods of absenteeism.

However, payment continuation in case of sick leave is organized differently in other countries indicating that our cost analysis is not directly transferable to other countries. Yet, the overarching message of the necessity of prevention strategies is very well transferable.

In countries in which employer payment continuation exceeds the duration of that in Germany, the matter may be even more pressing. For example, employers in the Netherlands have to maintain salary payment of sick employees for a duration of two years (i.e. 100% of salary during the first and 70% during the second year) (UWV 2020).

In the current study, we chose to explore the association of depressive symptom severity based on a self-report questionnaire with sick leave and associated costs. However, it may be assumed that similar or higher absenteeism and costs apply for persons with clinically confirmed depression.

Due to a limited identifiability of mental health conditions in the working environment, initiation of professional and adequate treatment may take a prolonged period of time. In this context, early prevention and promotion of (mental) health represent fields of action for all employers. The present study results emphasize the need for implementation, realignment or extension of professional work-site health promotion programs aiming (1) at the improvement and maintenance of employee health and (2) at the reduction of labour costs associated with depression related sick leave. Investment in such programs will, in addition to health benefits for employees, likely result in direct monetary benefit for employers, health insurances and the health security system in general.

## Supplementary Information

Below is the link to the electronic supplementary material.

Supplementary file1 (DOCX 25 KB)

## Data Availability

Source data are available upon request (The Robert Koch Institute, https://www.rki.de/EN/Content/Health_Monitoring/Public_Use_Files/public_use_file_content.html).
