# Supplementary material for: Absenteeism and associated labour costs according to depressive symptom severity in the German general population: why preventive strategies matter
Source: Int Arch Occup Environ Health. 2021 Oct 18;95(2):409–18. doi: 10.1007/s00420-021-01788-7 (PMC8794900; doi:10.1007/s00420-021-01788-7)
Supplement: Supplementary file 1 — Supplementary file1 (DOCX 25 KB) [file 420_2021_1788_MOESM1_ESM.docx]

| **Supplementary table S1 – Model building process for multivariable zero-inflated Poisson regression (ZIP) model exploring the association of depressive symptom severity with weeks of sick leave during 12 months** | | | | |
| --- | --- | --- | --- | --- |
| Independent variables in model | Exp (B) [95%CI] for main independent variable in count part of ZIP model, PHQ-8 (depressive symptoms, reference=no/minimal) | | LR chi2 | Difference in LR chi2 (p-value) |
| **Model 1:** PHQ-8 | Mild | 1.46 [1.41;1.51] | 2549.69 |  |
|  | Moderate | 2.30 [2.21;2.40] |  |  |
|  | Moderately severe | 2.82 [2.65;3.00] |  | Model 1 vs. model 2  1473.36 (0.00) |
|  | Severe | 4.38 [3.99;4.82] |  |  |
| **Model 2:** PHQ-8, age | Mild | 1.47 [1.43;1.52] | 4023.05 |  |
|  | Moderate | 2.27 [2.18;2.36] |  |  |
|  | Moderately severe | 2.87 [2.69; 3.05] |  | Model 2 vs. model 3  3.52 (0.06) |
|  | Severe | 4.17 [3.79;4.58] |  |  |
| **Model 3:** PHQ-8, age, sex | Mild | 1.47 [1.43;1.52] | 4026.57 |  |
|  | Moderate | 2.26 [2.17;2.36] |  |  |
|  | Moderately severe | 2.86 [2.68;3.04] |  | Model 3 vs. model 4*  443.59 (0.00) |
|  | Severe | 4.15 [3.77;4.56] |  |  |
| **Model 4:** PHQ-8, age, sex, BMI | Mild | 1.46 [1.41;1.51] | 4470.16 |  |
|  | Moderate | 2.19 [2.10;2.29] |  |  |
|  | Moderately severe | 2.72 [2.55;2.89] |  | Model 4 vs. model 5  316.87 (0.00) |
|  | Severe | 4.10 [3.73;4.50] |  |  |
| **Model 5:** PHQ-8, age, sex, BMI, smoking status | Mild | 1.43 [1.39;1.48] | 4787.03 |  |
|  | Moderate | 2.11 [2.03;2.21] |  |  |
|  | Moderately severe | 2.60 [2.44;2.77] |  | Model 5 vs. model 6  46.60 (0.00) |
|  | Severe | 3.87 [3.52;4.25] |  |  |
| **Model 6:** PHQ-8, age, sex, BMI, smoking status, marital status | Mild | 1.43 [1.39;1.48] | 4833.63 |  |
|  | Moderate | 2.10 [2.02;2.19] |  |  |
|  | Moderately severe | 2.58 [2.42;2.75] |  | Model 6 vs. model 7  557.17 (0.00) |
|  | Severe | 3.84 [3.49;4.22] |  |  |
| **Model 7:** PHQ-8, age, sex, BMI, smoking status, marital status, socio economic status | Mild | 1.41 [1.36;1.45] | 5390.80 |  |
|  | Moderate | 2.02 [1.93;2.10] |  |  |
|  | Moderately severe | 2.48 [2.33;2.64] |  | Model 7 vs. model 8  587.22 (0.00) |
|  | Severe | 3.62 [3.29;3.98] |  |  |
| **Model 8:** PHQ-8, age, sex, BMI, smoking status, marital status, socio economic status, number of comorbidities | Mild | 1.35 [1.31;1.40] | 5978.02 |  |
|  | Moderate | 1.88 [1.80;1.97] |  |  |
|  | Moderately severe | 2.27 [2.13;2.42] |  | Model 8 vs. model 9  7.76 (0.02) |
|  | Severe | 3.03 [2.75;3.33] |  |  |
| **Model 9** PHQ-8, age, sex, BMI, smoking status, marital status, socio economic status, number of comorbidities, Oslo-3 Social Support Scale | Mild | 1.36 [1.32;1.41] | 5985.78 |  |
|  | Moderate | 1.90 [1.82;1.99] |  |  |
|  | Moderately severe | 2.28 [2.14;2.43] |  | Model 8 vs. model 10*  13.78 (0.00) |
|  | Severe | 3.04 [2.75;3.35] |  |  |
| **Model 10** PHQ-8, age, sex, BMI, smoking status, marital status, socio economic status, number of comorbidities, support of/care for others | Mild | 1.35 [1.31;1.40] | 5991.80 |  |
|  | Moderate | 1.88 [1.80;1.96] |  |  |
|  | Moderately severe | 2.26 [2.12;2.41] |  | Model 8 vs. model 11**  286.59 (0.00) |
|  | Severe | 3.02 [2.74;3.33] |  |  |
| **Model 11**  PHQ-8, age, sex, BMI, smoking status, marital status, socio economic status, number of comorbidities, alcohol consumption | Mild | 1.36 [1.32;1.40] | 6264.61 |  |
|  | Moderate | 1.86 [1.78;1.94] |  |  |
|  | Moderately severe | 2.23 [2.09;2.38] |  |  |
|  | Severe | 2.94 [2.67;3.23] |  |  |
| *Of note: differences >20 in LR chi2 were considered relevant, statistical significance was assumed at p≤ 0.05 * despite the non-significant & non-relevant contribution of sex to the model-fit, it was kept for further analyses due to its potential role as effect-modifier*  *** since the addition of the variable for the* *Oslo-3 Social Support Scale resulted in a minor LR chi 2 difference (i.e. 7.76), the variable was discarded from the model and comparison with the previous model was performed*  **** since the addition of the variable for* support of/care for others *resulted in a minor LR chi 2 difference (i.e. 13.78), the variable was discarded from the model and comparison with the previous model was performed* | | | | |
